# The Relaxant Effects of Different Methanolic Fractions of *Nigella sativa* on Guinea Pig Tracheal Chains

**Published:** 2013-02

**Authors:** Rana Keyhanmanesh, Horeyeh Bagban, Hossein Nazemiyeh, Fariba Mirzaei Bavil, Mohammad Reza Alipour, Mehdy Ahmady

**Affiliations:** 1 Tuberculosis and Lung Research Centre, Tabriz University of Medical Sciences, Tabriz, Iran; 2 Department of Physiology, Faculty of Medicine, Tabriz University of Medical Sciences, Tabriz, Iran; 3 Research Centre for Pharmaceutical Nanotechnology, Faculty of Pharmacy, Tabriz University of Medical Sciences, Tabriz, Iran

**Keywords:** Guinea Pig, Methanolic Fractions, *Nigella sativa*, Relaxant Effect, Tracheal Chain

## Abstract

**Objective(s)**: In regard to the high incidence of asthma and the side-effects of the drugs used, finding novel treatments for this disease is necessary. Our previous studies demonstrated the preventive effect of *Nigella sativa* extract on ovalbumin-induced asthma. In addition, water-soluble substances of *N. sativa* extract and methanol fraction of this plant were responsible for the relaxant effect of this plant on tracheal chains of guinea pigs. Therefore, for the first time, in the present study, in order to identify main constituents of the methanolic extract, the relaxant effects of five different methanolic fractions (20%, 40%, 60%, 80%, and 100%) of *N. sativa* on tracheal chains of guinea pigs were examined.

**Materials and Methods:** The relaxant effects of four cumulative concentrations of each fraction (0.8, 1.2, 1.6, and 2.0 g%) in comparison with saline as negative control and four cumulative concentrations of theophylline (0.2, 0.4, 0.6, and 0.8 mM) were examined by their relaxant effects on precontracted tracheal chains of guinea pig by 60 mM KCl (group 1) and 10 µM methacholine (group 2).

***Results:*** In group 1, all concentrations of only theophylline showed significant relaxant effects but all concentrations of these methanolic fractions showed significant contractile effects compared with that of saline (*P<*0.001 to *P<*0.05). However, in group 2, all concentrations of theophylline and these methanolic fractions showed significant relaxant effects compared with that of saline (*P<*0.001 to *P<*0.05).

***Conclusion:*** These results showed a potent relaxant effect of 20% methanolic fractions from N. sativa on tracheal chains of guinea pigs that were higher than that of theophylline at the used concentrations.

## Introduction

Asthma is a global health problem affecting 300 million individuals of all ages, ethnic groups, and countries (1). Nowadays, many drugs are used for treating this illness. Although these drugs are effective, there are many side effects. Therefore, physicians try to find new drugs with fewer side effects. One important way for this purpose is studying the therapeutic effect of herbal medicine because some herbs have therapeutic effects without obvious side effects. 

Among the promising medicinal plants, *Nigella sativa*, a dicotyledone of the Ranunculaceae family, is an amazing herb with historical and religious background (2). This plant with green to blue flowers and small black seeds grows in temperate and cold climate areas. The seeds of *N. sativa* are the source of the active ingredients such as thymoquinone, monotropens-like P-cymene and α-pinene (3), nigellidine (4), nigellimine (5), and saponin (6).

Several therapeutic effects have been described for the seeds of *N. sativa* in medical books including anti-asthma and anti-dyspnea (7), hypotensive, anti-nociceptive, anti-fertility, anti-diabetic (8), anti-inflammatory, anti-oxidant, anti-microbial, anti-tumor, and immunomodulatory properties (9). There is evidence for relaxant effects of the volatile oil from this plant on different smooth muscle preparations including rabbit aorta (10), rabbit jejunum (11), and guinea pig isolated tracheal muscle (12). 

The results of recent studies also showed different pharmacological effects of *N. sativa* on guinea pig tracheal chains including relaxant and functional antagonistic effects on muscarinic receptors (13), inhibitory effect on histamine (H1) receptors (14), inhibitory effect on calcium channels (15), opening effect on potassium channels (16), and stimulatory effect on β-adronceptors (17). The antitussive effect of this plant on guinea pig (18) was also demonstrated.

**Figure 1:**
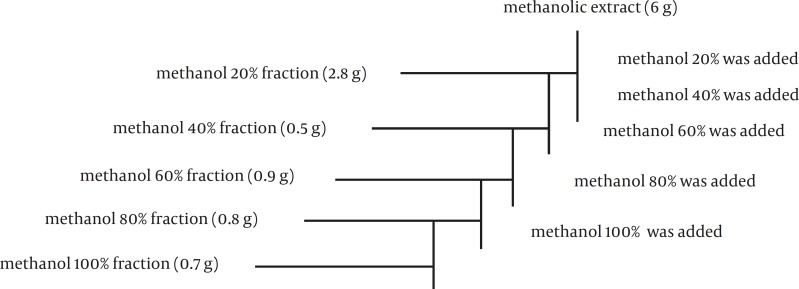
Fractionation of the methanolic extract from *N.* sativa

In addition, the preventive effects of hydro-ethanolic extract of *N. sativa* on lung pathological changes, tracheal responsiveness to methacholine and ovalbumin, cellular differentiation of bronchoalveolar lavage, and blood cytokines were also demonstrated (19, 20).

Our previous study in which the relaxant effects of hydro-ethanolic, macerated aqueous (MA) and lipid-free macerated aqueous (LFMA) extract of *N. sativa* were examined on guinea pig tracheal chains demonstrated that mainly water soluble substances of this plant were responsible for its relaxant effect (21). 

Moreover, the study of Boskabady *et al.* in 2008 showed that most concentrations of 3 different fractions of *N. sativa* (N-hexane, dichlorometane, and methanol fractions) had significant relaxant effects on guinea pig tracheal chains which was more potent for methanol fraction (22).

Therefore in the present study, the relaxant effects of five different methanolic fractions (20%, 40%, 60%, 80% and 100% methanolic extracts) on tracheal chains of guinea pigs were examined to identify main constituents of the methanolic extract of *N. sativa*,.

## Materials and Methods

*Plant and fractions*

*Methanolic fractions:* *N. sativa* was collected from north-east Iran and dried at room temperature in the absence of sunlight. The plant was identified by botanists in the herbarium of Ferdowsi University of Mashhad with specimen number of 293-0303-1. Methanolic fraction was prepared like the previous study (22). Briefly, n-hexane was added to 200 grams of the chopped, dried seeds and the solution was kept at room temperature for 10 hr. The solution was then separated and the solvent was dried. Then dichloromethane was added to the remaining powder at room temperature for 10 hr. After that, the solution was separated and the solvent was dried. Then methanol was added to the remaining powder at room temperature for 10 hr and the solution was separated and dried. In these manners, 31% lipid remaining of n-hexane fraction, 1% lipid remaining of dichloromethane fraction, and 7% lipid remaining of methanol fraction were obtained. 

After that, solid phase extraction (SPE) method was performed for preparing five different methanolic fractions (Figure 1). In this method, Sep-pac cartilages (10 gr- ODS, waters, Ireland) were used as solid phase and step gradients of methanol in water (methanol 20%, 40%, 60%, 80%, and 100%) were used as extraction solvents. In each stage, the solution was separated and dried. Then the 40 g% solution was prepared.

*Tissue preparation*

Guinea pigs (400-700 g, both sexes) were killed by a blow on the neck and tracheas were removed. Each trachea was cut into 10 rings (each containing 2-3 cartilaginous rings). All the rings were then cut open opposite the trachealis muscle, and sutured together to form a tracheal chain (21). Tissue was then suspended in a 20 ml organ bath (schuler organ bath type 809, March- Hugstetten, Germany) containing Krebs-Henseliet solution of the following composition (mM): NaCl 120, NaHCO_3_ 25, MgSO_4 _0.5, KH_2_PO_4_ 1.2, KCl 4.72, CaCl_2_ 2.5, and dextrose 11.

The Krebs solution was maintained at 37oC and gassed with 95% O_2_ and 5% CO_2_. Tissue was suspended under an isotonic tension of 1 g and allowed to equilibrate for at least 1 hr while it was washed with Krebs solution every 15 min. 

*Protocols*

The relaxant effects of different solutions were tested with two different experimental designs, (n= 6 for each group) as follows:

1. On tracheal chains contracted by 60 mM KCl (group 1 experiments). 

2. On tracheal chains contracted by 10 µM methacholine hydrochloride (Sigma Chemical Ltd, UK), (group 2 experiments).

The relaxant effects of four cumulative concentrations (0.8, 1.2, 1.6, and 2.0 g%) of five different methanolic fractions of *N. sativa* and theophylline anhydrous (Sigma Chemical Ltd, UK) (0.2, 0.4, 0.6, and 0.8 mM) as positive control, and normal saline (1 ml) as negative control were examined. In order to produce the first concentration of each fraction, 0.4 ml of 40 g% was added to a 20 ml organ bath and for other three concentrations; 0.2 ml of 40 g% was added to organ bath, respectively, three times. For theophylline, 0.2 ml of 20 mM theophylline solution was added to organ bath 4 times. The consecutive volumes were added to organ bath at 5 min intervals.

In each experiment, the effect of four cumulative concentrations of each fraction, theophylline or saline on contracted tracheal smooth muscle was measured after exposing tissue to each concentration of the solution for 5 min. A decrease in tone was considered to be a relaxant (bronchodilatory) effect and expressed as positive percentage change in proportion to the maximum contraction. An increase in tone was considered as a contractile (bronchoconstrictory) effect which was expressed as negative percentage change.

The relaxant effects in two groups of experiments were examined in two different series of tracheal chains. All of the experiments were performed randomly with 1 hr resting period of tracheal chains between each two experiments while washing the tissues every 15 min with Krebs solution. In all experiments, responses were amplified with amplifier (ML/118 quadribridge amp, March- Hugstetten, Germany) and recorded on powerlab (ML-750, 4 channel recorde r, March- Hugstetten, Germany).

*Statistical analysis*

All data were expressed as mean±SEM. Data of relaxant effects of different concentrations of each fraction were compared with the results of negative and positive control using paired t-test. The data of relaxant effects obtained in two groups of experiments were compared using unpaired t-test. The relaxant effects of different concentrations of five different fractions were compared with each other using one-way ANOVA. The relaxant effects of five fractions and theophylline were related to the concentrations using least square regression. Significance was accepted at *P<*0.05.

## Results

*Relaxant (bronchodilatory) effect*

**Figure 2:**
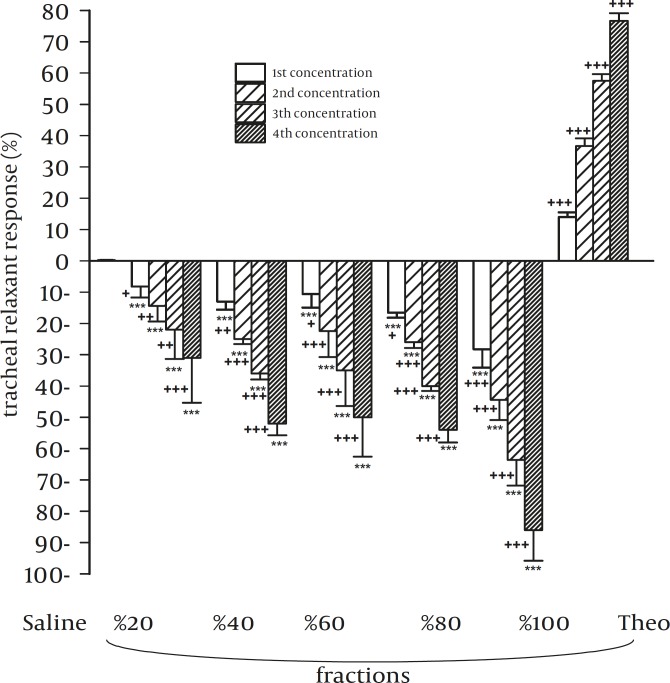
Relaxant effects of five different methanolic extracts from Nigella sativa in comparison with negative control (saline) and positive control (theophylline) in group 1 experiments (contracted tracheal chains with 60 mM KCl, n=6) Statistical differences in the relaxant effects of different concentrations of different methanolic fractions and theophylline vs. that of saline. ns: non-significant difference, +: *P*<0.05, ++: *P*<0.01, +++: *P*<0.001. Statistical differences in the relaxant effects of different concentrations of different methanolic fractions vs. that of theophylline: NS: non-significant difference, *: *P*<0.05, **: *P*<0.01, ***: *P*<0.001

In group 1 experiments, all concentrations of only theophylline showed significant relaxant effects compared to that of saline (*P<*0.001 for all concentrations). All concentrations of different methanolic extracts showed significant contractile effects compared with that of saline in this group (*P<*0.001 to *P<*0.05) (Figure 2).

**Figure 3:**
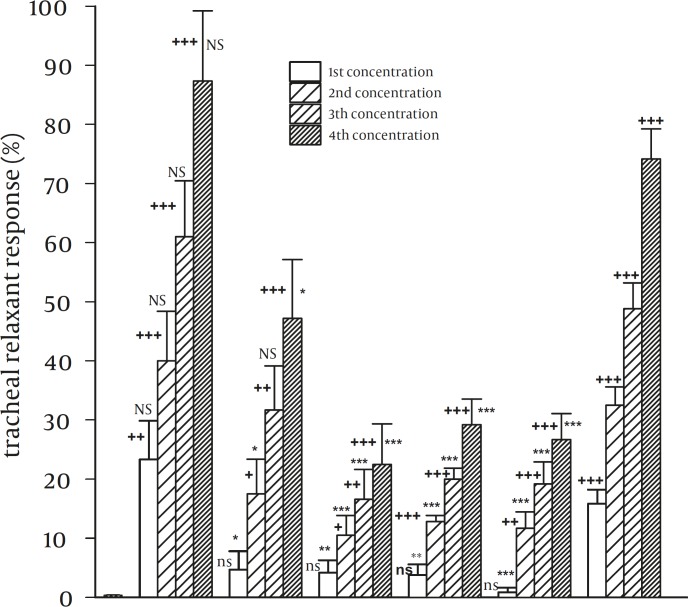
Relaxant effects of five different methanolic extracts from Nigella sativa in comparison with negative control (saline) and positive control (theophylline) in group 2 experiments (contracted tracheal chains by 10 µM methacholin, n=6) Statistical differences in the relaxant effects of different concentrations of different methanolic fractions and theophylline vs. that of saline. ns: non-significant difference, +: *P*<0.05, ++: *P*<0.01, +++: *P*<0.001. Statistical differences in the relaxant effects of different concentrations of different methanolic fractions vs. those of theophylline: NS: non-significant difference, *: *P*<0.05, **: *P*<0.01, ***: *P*<0.001

In group 2 experiments, all concentrations of theophylline and 20% methanolic extract and three last concentrations of other different methanolic extracts (40%, 60%, 80%, and 100%) showed significant relaxant effects compared with that of saline (*P<*0.001 to *P<*0.05). However, the first concentrations of these extracts (40%, 60%, 80%, and 100% methanolic extracts) showed non-significant relaxant effects on tracheal chains (Figure 3).

*Comparison of relaxant effects of theophylline with different extracts*

In group 1 and 2, relaxant effects of all concentrations of all methanolic fractions (except all concentrations of 20% methanolic extract and the third concentration of 40% methanolic extract in group 2) were significantly less than those of theophylline (*P<*0.001 to *P<*0.05). However, the relaxant effects of all concentrations of 20% methanolic extract were non-significantly higher than those of theophylline (Figures 1 and 2).

*Comparison of relaxant effects of different extracts*

In group 1, the contractile effects of all concentrations of 20% and 40% methanolic extracts, the first and the last concentrations of 60% methanolic extract and three higher concentrations of 80% methanolic extract were significantly lower than those of 100% methanolic extract (*P<*0.001 to *P<*0.05). The most potent contractile effect was seen for 100% methanolic extract and on the contrary, 20% methanolic extract showed the lowest contractile effect in comparison with others.

In group 2, the relaxant effects of all concentrations of 20% methanolic extract were significantly higher than those of others (*P<*0.001 to *P<*0.05). There is no significant difference between the relaxant effects of different concentrations of four other extracts (40%, 60%, 80%, and 100%) (Figures 1 and 2).

*Comparison of the relaxant effect between two groups of experiments*

**Figure 4:**
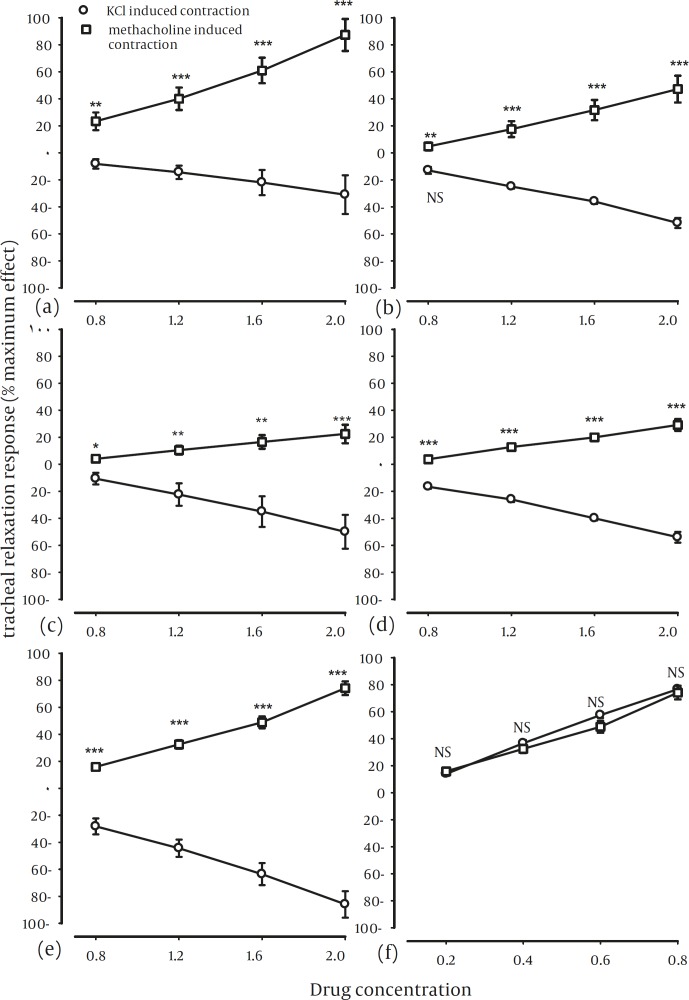
Concentration response curves of the relaxant effect of 20% methanolic (a), 40% methanolic (b), 60% methanolic (c), 80% methanolic (d), 100% methanolic (e) extracts from *Nigella sativa* and theophylline (f) in two groups of experiments. Group 1: KCl induced contraction of tracheal chains (o) and group 2: methacholine induced contraction of tracheal chains (n=6 for both groups). Statistical differences in the relaxant effect of different concentrations of each fraction between group 1 with those of group 2: NS: non-significant difference, ***:*P*<0.001. The concentration unit for fractions was g%, and for theophylline, mM

The relaxant effects of different concentrations of different methanolic extracts were significantly greater in group 2 compared with group 1 experiments (*P<*0.001). However, there was no significant difference in the relaxant effects of different concentrations of theophylline between two groups (Figure 4).

*Correlation between concentrations of solution and their relaxant effects*

There were significant positive correlation between relaxant effects and concentrations for all extracts in group 2 and for theophylline in both groups (*P<*0.001 to *P<*0.01). However, the correlation between the relaxant effects and concentrations for all extracts (except 20% methanolic extract) in group 1 were significantly negative (*P<*0.001 to *P<*0.01) (Table 1).

## Discussion

In this study, the relaxant (bronchodilatory) effects of five different methanolic fractions (20%, 40%, 60%, 80%, and 100%) from *N. sativa* were compared with saline as negative control and theophylline as positive control. In group 1 experiments (contracted tracheal chains by KCl), all concentrations of only theophylline showed significant relaxant effects compared with that of saline. All concentrations of methanolic fractions showed significant contractile effects compared with that of saline in this group. Less contractile effect was seen for 20% methanolic fraction. In group 2 experiments (contracted tracheal chains by methacholine), all concentrations of theophylline and 20% methanolic fractions and most concentrations of other methanolic fractions showed significant relaxant effects compared with that of saline. The relaxant effects of all concentrations of methanolic fractions except 20% methanolic fraction were significantly less than those of theophylline. However, the relaxant effects of all concentrations of 20% methanolic fraction were non-significantly higher than those of theophylline in group 2. There were no significant differences in the relaxant effect of different concentrations of theophylline between the two groups. There were significant positive correlations between the relaxant effects and concentrations for all fractions in group 2 and for theophylline in both groups. 

The bronchodilatory effect seen for methanolic fractions of *N. sativa* in this study was in accordance to the results of previous study (22) and might be produced due to several different mechanisms such as inhibitory effect on muscarinic receptors (16), potassium channels opening effect (23), and antioxidant effect as described in previous studies (24- 26). Although lack of the relaxant effect of different methanolic fractions from *N. sativa* on tracheal chains contracted by KCl (group 1) did not suggest an inhibitory effect of this plant on calcium channels of guinea pig tracheal chain which is in agreement with previous studies (16, 21, 22). 

In this investigation, the 20% methanolic fraction of *N. sativa* showed more relaxant effect compared with others. The exact reason for the most relaxant effect of this fraction is uncertain to us and should be clarified in future studies but some suggested reasons are as follows: 

First, as through solid phase extraction (SPE) method for preparing different methanolic fractions (as mentioned in materials and methods part), we used step gradients of methanol in water. The less relaxant effects of four different fractions in comparison with 20% methanolic fraction may be due to lack of relaxant constituents which were extracted in the first step in 20% methanolic fraction. Second, it may be due to the presence of the solvents in different fractions although almost all were dried in preparing the fractions as mentioned previously.

As this was the first investigation to determine the main effective constituent of methanolic fraction of this plant causing tracheal muscle relaxation in order to find novel treatment for cases with increased airway resistance such as asthmatics, it is proposed to assess the relaxant effects of different methanolic fractions on normal guinea pig tracheal muscle. The evaluation of the relaxant effect of this extract on tracheal chains of asthmatic guinea pigs is suggested for future studies and more studies are required for clearing the exact mechanism(s) and the effective substances.

**Table 1:** Correlation (r) between the relaxant effects of five different methanolic extracts (20%, 40%, 60%, 80%, and 100% methanolic fractions) from *Nigella sativa* and theophylline with their concentrations in two groups of experiments

Different solutions	20% Methanolic extract		40% Methanolic extract		60% Methanolic extract		80% Methanolic extract		100% Methanolic extract		Theophylline	
	r	*P-*value	r	*P-*value	r	*P-*value	r	*P*-value	r	*P*-value	r	*P*-value
Group 1	-0.423	NS	-0.938	*P<*0.001	-0.602	*P<*0.01	-0.940	*P<*0.001	-0.810	*P<*0.001	0.978	*P<*0.001
Group 2	0.752	*P<*0.001	0.708	*P<*0.001	0.545	*P<*0.01	0.849	*P<*0.001	0.793	*P<*0.001	0.921	*P<*0.001

## Conclusion

The results of the present study showed a potent relaxant effect of methanolic fractions from *N. sativa* on tracheal chains of guinea pigs. The most potent relaxant effect was seen for 20% methanolic fraction that was non-significantly higher than that of theophylline at concentrations used.
